# Association Between Cerebrospinal Fluid Biomarkers and Age-related Brain Changes in Patients with Normal Pressure Hydrocephalus

**DOI:** 10.1038/s41598-020-66154-y

**Published:** 2020-06-04

**Authors:** Foad Taghdiri, Melisa Gumus, Musleh Algarni, Alfonso Fasano, David Tang-Wai, Maria Carmela Tartaglia

**Affiliations:** 1 Tanz Centre for Research in Neurodegenerative Diseases, University of Toronto, 60 Leonard avenue, Toronto, ON M5T 2S8 Canada; 2 Department of medicine, Division of Neurology, University of Toronto, Toronto, ON Canada; 3 Edmond J. Safra Program in Parkinson’s Disease, Morton and Gloria Shulman Movement Disorders Clinic, Toronto Western Hospital, UHN, Toronto, Ontario, Canada. Division of Neurology, University of Toronto, Toronto, Ontario Canada; 4 Krembil Brain Institute, Toronto, Ontario Canada

**Keywords:** Neuroscience, Cognitive ageing, Cognitive neuroscience, Diseases of the nervous system

## Abstract

Our study aimed to: 1)investigate the diagnostic utility of CSF Aβ42, t-tau, and p-tau to differentiate normal-pressure-hydrocephalus(NPH) from Alzheimer’s-disease(AD) and normal-controls; and 2)investigate if age and ventricular size affect the levels of CSF biomarkers in NPH patients. We recruited 131 participants: (a)Suspected-NPH: 72 with ventriculomegaly and clinical symptoms of NPH. These participants were then divided into two groups of 1)Probable-NPH (N = 38) and 2)Unlikely-NPH (N = 34) based on whether participants experienced gait improvement after removal of a large amount of CSF; (b)AD group: 30 participants with CSF biomarkers and cognitive symptoms consistent with AD; (c)Control-group: 29 participants who were cognitively and functionally normal. Lower levels of CSF Aβ42 and p-tau were observed in the probable-NPH compared to the normal controls(444.22 ± 163.3 vs. 1213.75 ± 556.5; and 26.05 ± 9.2 vs. 46.16 ± 13.3 pg/mL; respectively). Lower levels of CSF p-tau and t-tau were found in the probable-NPH compared to the AD(26.05 ± 9.2 vs. 114.95 ± 28.2; and 193.29 ± 92.3 vs. 822.65 ± 311.5 pg/mL; respectively) but the CSF-Aβ42 was low in both the probable-NPH and AD. CSF-Aβ42 correlated with age and Evans-index only in the probable-NPH(r = 0.460, p = 0.004; and r = −0.530, p = 0.001; respectively). Our study supports the hypothesis that age-related atrophy results in better Aβ42 clearance in the CSF because of the increase in the interstitial space.

## Introduction

Normal pressure hydrocephalus (NPH) is a syndrome associated with enlarged ventricles without marked elevation in cerebrospinal fluid (CSF) pressure^1^. Clinical symptoms include gait and balance impairment, cognitive deficits, and urinary urgency/incontinence^1^. In both NPH and Alzheimer’s disease (AD) decreased CSF levels of the amyloid β−42 (Aβ42) have been found; however, in contrast to AD, total tau (t-tau) and phospho-tau (p-tau) levels are not increased in NPH cases^2,3^. It has been hypothesized that the low Aβ42 in the presence of low t-tau and p-tau is due to the decrease in interstitial space that precludes amyloid precursor protein (APP) fragments and tau proteins from being effectively cleared by CSF and consequently the levels of these proteins decrease in the CSF^4^. This hypothesis was based on two major observations: 1) low levels of all APP fragments (i.e., Aβ38, Aβ40, Aβ42, sAPPα, and sAPPβ) in CSF obtained both by lumbar and ventricular methods of patients with NPH compared to health controls, which all increased back to normal after shunting^1^ and 2) Aβ clearance from the interstitial fluid is increased during sleep when the size of interstitial space increases by 60%^5^.

The aims of our study were to: 1) investigate the diagnostic utility of CSF Aβ42, t-tau, and p-tau to differentiate NPH from AD and normal controls; and 2) investigate if age and ventricular size affect the levels of these CSF biomarkers in NPH patients. Answers to these questions would improve understanding of CSF Aβ42, t-tau, and p-tau production/clearance and facilitate relating CSF biomarkers to patient characteristics.

## Results

### Differences in CSF biomarkers between the groups

**Figure 1:**
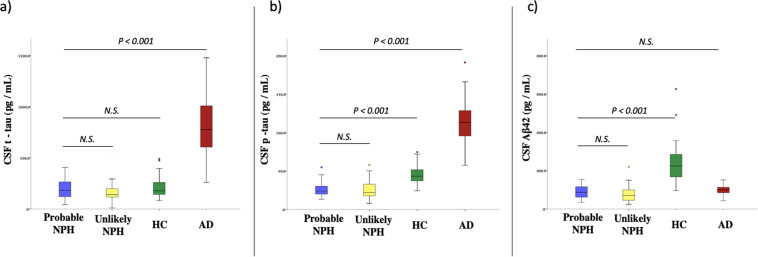
Comparing the levels of CSF biomarkers: (**a**) t-tau, (**b**) p-tau, and (**c**) Aβ42 between the groups (i.e., Probable NPH, unlikely NPH, Normal controls (NC), and Alzheimer’s disease (AD).

Average age of the patients in the probable and unlikely NPH groups was not significantly different (73.49 ± 6.3 vs 70.54 ± 7.74 years old; p = 0.13). CSF levels of Aβ42, t-tau, and p-tau were not significantly different between the probable and unlikely NPH groups (444.22 ± 163.3 vs. 392.61 ± 213.6 pg/mL, p = 0.825; 193.29 ± 92.3 vs. 158.46 ± 92.9 pg/mL, p = 0.514; and 26.05 ± 9.2 vs. 24.67 ± 11.4 pg/mL, p = 0.994; respectively). However, significantly lower levels of the CSF Aβ42 and p-tau were observed in the probable NPH group compared to the normal controls (444.22 ± 163.3 vs. 1213.75 ± 556.5 pg/mL, p < 0.001; and 26.05 ± 9.2 vs. 46.16 ± 13.3 pg/mL, p < 0.001; respectively). When CSF Aβ42, t-tau and p-tau were compared between the probable NPH group and AD group, lower levels of CSF p-tau and t-tau were found in the probable NPH group compared to the AD group (26.05 ± 9.2 vs. 114.95 ± 28.2 pg/mL, p < 0.001; and 193.29 ± 92.3 vs. 822.65 ± 311.5 pg/mL; respectively) but the mean CSF Aβ42 was low in both the probable NPH and AD group (444.22 ± 163.3 vs 493.20 ± 139.8 pg/mL, p = 0.703). Figure 1 and Table 1 summarize the levels of CSF biomarkers between the groups.

**Table 1:** Groups description*.

	Suspected NPH		AD Group	Control Group
	Probable NPH	Unlikely NPH		
N	38	34	30	29
Age (years)	73.49 ± 6.3	70.54 ± 7.74	74.06 ± 7.7	71.9 ± 5.4
Abnormal Cognitive Assessment score^†^	38/38	34/34	30/30	0/29
CSF Aβ42 (pg/mL)	444.22 ± 163.3	392.61 ± 213.6	493.20 ± 139.8	1213.75 ± 556.5
CSF t-tau (pg/mL)	193.29 ± 92.3	158.46 ± 92.9	822.65 ± 311.5	216.27 ± 107.3
CSF p-tau (pg/mL)	26.05 ± 9.2	24.67 ± 11.4	114.95 ± 28.2	46.16 ± 13.3

*Mean ± SD; ^†^Number of participants in each group with abnormal cognitive (i.e., MoCA, or TorCA) scores.

### PCA results

**Figure 2:**
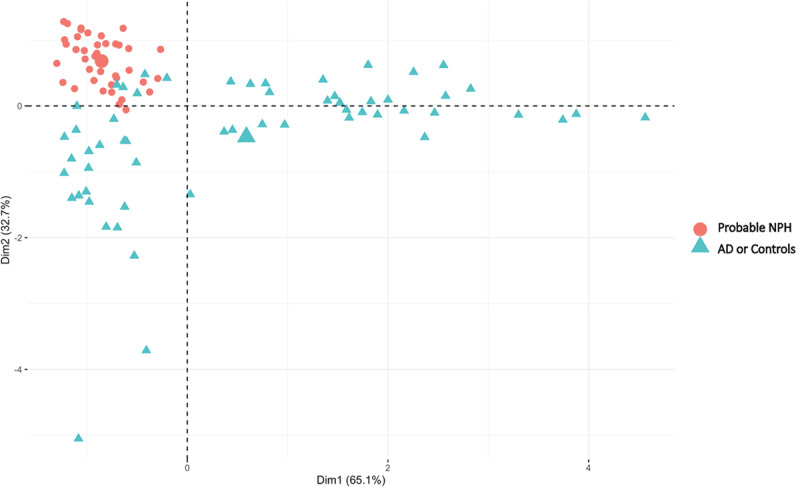
PCA results. Red dots represent probable NPH patients while blue dots represent AD patients or controls.

Although the CSF biomarkers could not differentiate probable NPH from unlikely NPH, PCA was able to differentiate the probable NPH group from the AD and normal controls groups (Fig. 2). Initial eigenvalues indicated that the first three components explained 65%, 33%, and 2% of the variance, respectively. We did not eliminate any of the factors from the PCA analysis since p-tau and t-tau showed factor loading of about 0.7 in the first component while Aβ42 had -1.0 factor loading in the second component. We then used the first 2 principal components from the PCA in the SVM to investigate the model predictions in separating the patient groups based on their t-tau, p-tau and Aβ42 profiles. The SVM predicted the patient category for each individual with a 92% accuracy (95% CI = 80%, 97%; p < 0.001) resulting in a sensitivity and specificity of 90% and 98%, respectively.

### Relationship between the CSF biomarkers and age in the probable and unlikely NPH groups

**Figure 3:**
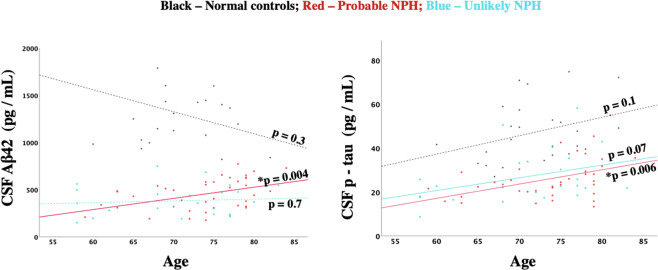
Relationship between CSF biomarkers and age in the probable (red) and unlikely (blue) NPH groups and controls (black).

Age was significantly correlated with the levels of CSF Aβ42 and p-tau in the probable NPH group (r = 0.460, p = 0.004; and r = 0.444, p = 0.006; respectively) but not in the unlikely NPH group (r = 0.09, p = 0.674; and r = 0.381, p = 0.066; respectively) or in the normal controls (r = −0.221, p = 0.289; and r = 0.330, p = 0.107; respectively) (Fig. 3). Age was not correlated with the levels of CSF t-tau in either of the groups (i.e., probable NPH, unlikely NPH, and normal controls) (r = 0.298, p = 0.073; r = 0.165, p = 0.441; r = 0.271, p = 0.191; respectively).

### Relationship between the CSF biomarkers and Evans index in the probable and unlikely NPH groups

**Figure 4:**
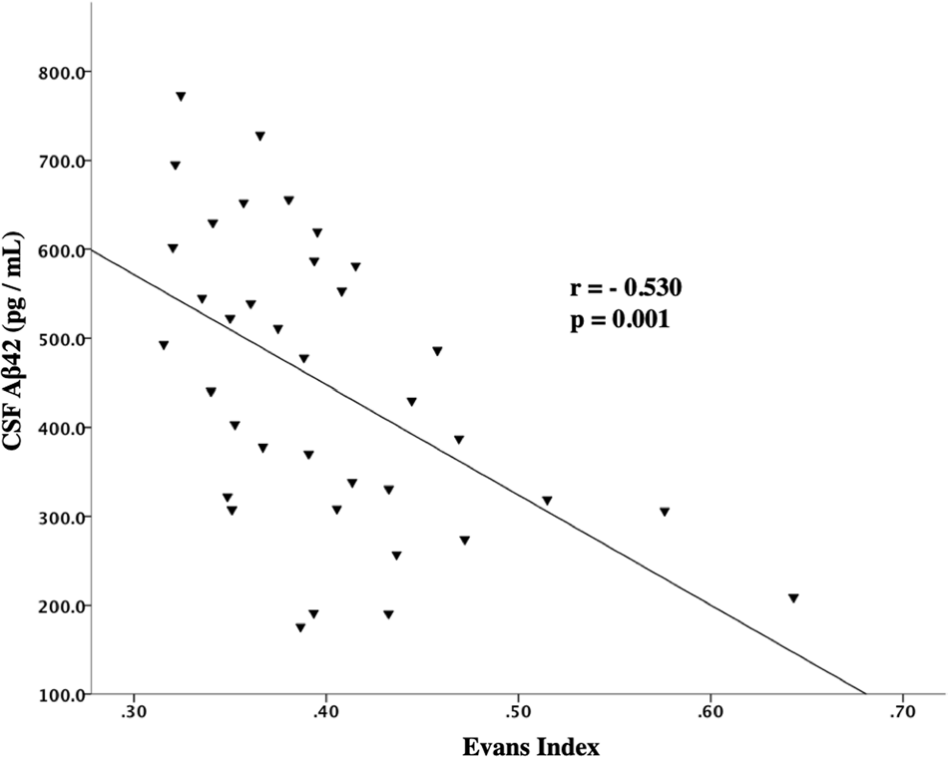
Correlation between CSF Aβ42 levels and Evans index in the probable NPH group.

Evans index was negatively correlated with the levels of CSF Aβ42 (r = −0.530, p = 0.001) in the probable NPH group (Fig. 4). However, no statistically significant correlation was found between the Evans index and the levels of CSF p-tau and t-tau in this group (r = −0.229, p = 0.172; and r = −0.049, p = 0.775; respectively). In the unlikely NPH group, none of the CSF Aβ42, p-tau, and t-tau levels were significantly correlated with the Evans index (r = −0.016, p = 0.938; r = 0.052, p = 0.799; and r = 0.257, p = 0.204; respectively).

### Linear regression model

As mentioned above, the levels of CSF Aβ42 were significantly correlated with both age and Evans index in the probable NPH group. To further assess these relationships, we ran a linear regression analysis entering the CSF Aβ42 as a dependent variable and age and Evans index as two independent variables. This model could explain 32.6% of the variability seen in the levels of CSF Aβ42 (R = 0.571, p = 0.001) and showed that Evans index is contributing to the levels of CSF Aβ42 independent of age (standardized coefficient beta = −0.398, p = 0.022) in the probable NPH group.

## Discussion

The results of this study revealed lower levels of CSF Aβ42 and p-tau in the probable NPH group compared to the controls. The mean level of CSF Aβ42 was similar between the probable NPH and AD groups. As our PCA results showed, including CSF Aβ42, p-tau, and t-tau allowed differentiation of probable NPH from controls and patients with AD (Fig. 2). However, these CSF biomarkers were not significantly different between the probable and unlikely NPH so could not be used to differentiate between these two groups.

The PCA/SVM results (Fig. 2) revealed that CSF Aβ42, p-tau, and t-tau, could be used to differentiate probable NPH from AD and normal controls with a reasonable sensitivity and specificity (i.e. 90% and 98%, respectively). However, it should be noted that although a pattern of low CSF Aβ42 level in the presence of low to normal p-tau and t-tau levels in the context of clinical and radiological findings consistent with NPH can increase the likelihood of this disease, it is not pathognomonic. Levels of CSF Aβ42, p-tau, and t-tau failed to differentiate the probable from unlikely NPH in the present study. Although not clear, one possibility is that other comorbidities in the unlikely NPH group are confounding the results.

Interestingly, in the probable NPH group, CSF Aβ42 was significantly correlated with age and inversely correlated with size of the ventricles, as measured using Evans index. These findings are consistent with the hypothesis that in the setting of NPH, the interstitial space between neurons is decreased and consequently clearance of Aβ42 into the CSF is impaired^4^. Traditionally, the Evans index has been used as an indirect surrogate marker of ventricular volume in CT and MRI^6,7^. Larger ventricular volumes, thus higher values of Evans index, in NPH may be associated with less interstitial space. This decreased interstitial space impairs the clearance of Aβ42 into CSF and may explain the negative correlation observed between the CSF Aβ42 and Evans index. On the other hand, aging is associated with brain atrophy which increases interstitial space and thus allows better clearance of the Aβ42 from the brain. In other words, the natural process of aging and brain atrophy can potentially help with the normalization of the CSF biomarkers in patients with NPH. In addition, these results suggest that patients’ characteristics such as their age and the amount of ventriculomegaly should be taken into account before interpreting the CSF biomarkers.

As mentioned above, investigating levels of CSF Aβ42, p-tau, and t-tau alone does not suffice to differentiate the probable from unlikely NPH group. However, our results also revealed that CSF Aβ42 is significantly related to the patients’ age only in the probable NPH and not in the unlikely NPH group. This raises the question whether longitudinal follow up of CSF Aβ42 levels rather than measuring them at a single timepoint, may be better for selecting patients with probable NPH. However, more longitudinal studies with large sample sizes are necessary to further investigate this hypothesis. In addition, in this study, we did not investigate the potential causes for the observed decrease in CSF Aβ42 in the probable NPH group (e.g., formation of amyloid plaques, dilution effect, or less production of Aβ42) and more studies are required to investigate this further. Also, it should be noted that as mentioned above, approximately 40 mL of CSF was collected from each NPH participant. This is a relatively large amount of CSF and may potentially affect the biomarker concentrations. However, large amount CSF collection is common in NPH assessment^8^. The other potential limitation of our study is that we did not measure other possible CSF biomarkers such as Aβ40 or neurofilament light chain. Measuring these biomarkers may potentially help better understand NPH pathophysiology.

In conclusion, our study showed that the mean level of CSF Aβ42 was similar between the probable NPH and AD groups and lower than that of normal controls while both p-tau and t-tau levels were significantly lower in probable NPH compared to AD. Also, probable NPH patients with higher Aβ42 values tended to be older in our study which supports the hypothesis that age-related atrophy results in better Aβ42 clearance in the CSF because of the increase in the interstitial space. Finally, given the association between age and levels of CSF Aβ42 in the probable NPH group, we proposed a hypothesis that following up on the levels of CSF biomarkers longitudinally is a stronger biomarker for the diagnosis of probable NPH compared to a single measurement of these biomarkers.

## Methods

### Participants

A total of 131 subjects were included in this study:

(a) Suspected NPH group: 72 patients (age [mean ± SD], 73.04 ± 6.5 years) with ventriculomegaly on MRI brain or CT head with clinical symptoms of NPH (i.e., cognitive impairment, balance/gait impairment, and urinary incontinence) were recruited by three neurologists (MCT, DTW, and AF) through Toronto Western Hospital, University Health Network (UHN), Toronto, Canada. All patients underwent lumbar puncture (LP) and all with a normal opening pressure^6^. Patients with suspected NPH group were divided into two groups based on the criteria previously described by Relkin *et al*.^6^: 1) Probable NPH: 38 patients with clinical symptoms and radiological findings consistent with NPH who experienced an improvement in their gait after removal of a large amount of CSF (35–40 ml). Large amount of CSF removal caused clinically significant improvement in this group’s patients’ gait. Patients in this group were not suffering from any other neurological, psychiatric, or medical conditions that could potentially explain their presenting symptoms; 2) Unlikely NPH: 34 patients with clinical symptoms similar to the previous group but large amount of CSF removal did not significantly improve their gait impairment, i.e. little or no change in speed or stride length.

(b) AD group: included 30 patients (age, 74.06 ± 7.7 years) with CSF biomarkers consistent with AD^9,10^.

(c) Control group: 29 subjects who were cognitively and functionally normal including gait and neuroimaging but some had a history of depression but no evidence of AD on CSF biomarkers.

### Cognitive assessments

As part of the clinical evaluation all participants underwent a cognitive assessment. However, different tests (i.e., Montreal Cognitive Assessment (MoCA)^11^, Toronto Cognitive Assessment (TorCA)^12^) were used to assess the cognitive status.

### Standard protocol approvals and patient consents

The University Health Network’s Research Ethics Board approved the study. Written informed consent was obtained from all participants or their legally authorized representatives, in case the participant was not competent to consent, before participating in the study. All methods were carried out in accordance with relevant guidelines and regulations.

### CSF analysis

All subjects underwent a lumbar puncture (LP) that was performed according to Alzheimer’s Disease Neuroimaging Initiative (ADNI) protocol^13^. Approximately, 40 mL of CSF was collected from each NPH participant by allowing CSF to drip into the collecting tubes (gravity drip). After CSF was collected in polypropylene tubes, it was transported to the adjacent laboratory within 30 minutes, aliquoted, and stored at -80 °C. A sandwich ELISA was used to measure concentrations of Aβ42 (Innotest β-amyloid (1–42), Fujirebio), p-tau (Innotest phospho-tau (181p), Fujirebio), and t-tau (Innotest hTAU-Ag, Fujirebio) following the manufacturer’s instruction^14,15^. All analyses were performed at the Tanz Centre for Research in Neurodegenerative Diseases (Toronto, Ontario) by one operator (FT). All samples were measured in duplicate and repeated if the difference between individual optic density (OD) values was greater than 20%. In addition to the ready-to-use calibrators (CAL) and Run Validation Controls (RVC) which were part of the Fujirebio Innotest assay kits, internal controls were also included in each run. After calculating the mean absorbance for the CAL, RVC, and unknown CSF samples, a sigmoidal 4-parameter curve fitting was used to determine the corresponding concentrations. CSF biomarkers were considered consistent with AD diagnosis if p-tau > 68 pg/ml and Aβ42 to t-tau index (ATI) < 0.8^9,10^.

### Evans index

Evans index was calculated for the subjects in the suspected NPH group using their axial Fluid-attenuated inversion recovery (FLAIR) magnetic resonance images (MRI) or axial computed tomography (CT) images when MRI was not available. The ratio of the maximum width of the frontal horns of the lateral ventricles to the maximal internal diameter of the skull at the same slice of the axial MRI or CT images was used to calculate this index^16^.

### Principle component analysis (PCA)

We constructed a Support Vector Machine (SVM) to separate the probable NPH patients from AD and controls based on individual levels of CSF Aβ42, t-tau and p-tau. SVM is a powerful supervised learning algorithm that can be trained on a subset of data and test its predictions on the rest of the testing data. First, a Principle Component Analysis (PCA) was used to visualize the data and compute composite scores for Aβ42, t-tau and p-tau for each patient. The first two components of PCA were then used in the SVM for determining whether the linear combination of CSF Aβ42, t-tau, p-tau levels separated probable NPH patient from the other two groups (i.e., AD and controls). The SVM was designed with a linear kernel for the classification, and we used 8-fold cross validation that was implemented in the function. PCA in this process was not used to select the significant factors, but rather utilized to create composite variables as an input for SVM. For this step, all data was scaled and processed using R studio^17^.

### Statistical analysis

Statistical analyses were conducted using SPSS software (SPSS Inc. v. 24). One-way analysis of variance (ANOVA) was used to compare the CSF biomarkers (i.e., Aβ42, t-tau and p-tau) among the groups (i.e., probable NPH, unlikely NPH, AD, NC). Dunnett-T3 post hoc analysis^18^ was performed to compare the probable NPH group with each of the 3 other groups. The Pearson correlation coefficient was used for correlations. Statistical significance level was set at p < 0.05.

## Data Availability

All data can be shared at the request of other investigators.
